# Monofunctional Heme-Catalases

**DOI:** 10.3390/antiox11112173

**Published:** 2022-11-02

**Authors:** Wilhelm Hansberg

**Affiliations:** Departamento de Biología Celular y del Desarrollo, Instituto de Fisiología Celular, Universidad Nacional Autónoma de México (UNAM), Mexico City 04510, Mexico; whansberg@ifc.unam.mx; Tel.: +52-55-5622-5655; Fax: +52-55-5622-5630

**Keywords:** molecular dynamics, gate valve mechanism, reactive compound I, catalase new functions, heme *d*, singlet oxygen, proximal Tyr covalent bond, origin of C-terminal domain, Hsp31, molecular chaperone

## Abstract

The review focuses on four issues that are critical for the understanding of monofunctional catalases. How hydrogen peroxide (H_2_O_2_) reaches the active site and outcompetes water molecules to be able to function at a very high rate is one of the issues examined. Part of the answer is a gate valve system that is instrumental to drive out solvent molecules from the final section of the main channel. A second issue relates to how the enzyme deals with an unproductive reactive compound I (Cpd I) intermediate. Peroxidatic two and one electron donors and the transfer of electrons to the active site from NADPH and other compounds are reviewed. The new ascribed catalase reactions are revised, indicating possible measurement pitfalls. A third issue concerns the heme *b* to heme *d* oxidation, why this reaction occurs only in some large-size subunit catalases (LSCs), and the possible role of singlet oxygen in this and other modifications. The formation of a covalent bond between the proximal tyrosine with the vicinal residue is analyzed. The last issue refers to the origin and function of the additional C-terminal domain (TD) of LSCs. The TD has a molecular chaperone activity that is traced to a gene fusion between a Hsp31-type chaperone and a small-size subunit catalase (SSC).

## 1. Introduction

Hydrogen peroxide is produced in most cells, both in aerobes and anaerobes. To a certain extent, most anaerobes are aerotolerant and express antioxidant enzymes. The main source of H_2_O_2_ in aerobic cells is a disproportion of superoxide (O_2_^•−^). Superoxide is formed mainly in the respiratory chain and is the product of some oxidases, such as NADPH oxidase and xanthine oxidase. Autooxidation of heme proteins, such as hemoglobin, myoglobin, and cytochrome P450, can also form O_2_^•−^. Superoxide is dismutated by superoxide dismutases (SODs), which are abundant and very efficient enzymes (2 × 10^9^ M^−1^ s^−1^) [1]. SODs are present in different cellular compartments and also outside cells. Thus, H_2_O_2_ is produced in various sites, inside and outside cells, and is not homogeneously distributed in the cytoplasm [2]. H_2_O_2_ has important functions in animals, such as synthesis of thyroid hormone, sperm maturation and capacitation, formation of the fertilization membrane, extracellular matrix crosslinks, and production of hypochlorous acid by myeloperoxidase, among others. Additionally, H_2_O_2_ is considered a signal molecule in organisms. Lignin degradation by fungi is another important function of H_2_O_2_.

Although a strong oxidant, H_2_O_2_ is a mildly reactive compound and does not react with most cellular components, such as NAD(P)H, proteins, nucleic acids, and lipids. It reacts poorly with glutathione, ascorbate, pyruvate, or α-ketoglutarate. However, when intracellular O_2_^•−^ and H_2_O_2_ increase to micromolar levels, harmful effects can occur, not because O_2_^•−^ and H_2_O_2_ are toxic per se, but because both can react with the [Fe-S] centers of enzymes, thereby inhibiting these enzymes and liberating iron from them. The toxic effect of H_2_O_2_ is mainly related to free iron: H_2_O_2_ reacts with Fe^II^ to form the highly reactive hydroxyl radical (Fenton reaction). O_2_**^•−^** reduces Fe^III^ to form Fe^II^ and O_2_. Thus, O_2_^•−^ acts as an enhancer of the Fenton reaction producing a continuous cycle of hydroxyl radical generation unless the iron is chelated by a compound in an unreactive manner. Furthermore, some spontaneous reactions with H_2_O_2_ can give rise to singlet oxygen. Both hydroxyl radical and singlet oxygen react with almost any nearby molecule causing damage to proteins, nucleic acids, and lipids.

Different enzymes maintain the intracellular H_2_O_2_ concentration at a low level: peroxiredoxins keep H_2_O_2_ at nanomolar levels; however, in eukaryotes these enzymes are inhibited at approximately 10 µM H_2_O_2_ [3,4]. At this H_2_O_2_ concentration and up to 1 mM, catalase-peroxidase [5,6,7,8] and other peroxidases are active; at mM H_2_O_2_ concentrations, catalases become the principal H_2_O_2_-disposing enzymes [9,10,11]. Thus, catalases are important when there is oxidative stress.

Different enzymes exhibit catalase activity (E.C. 1.11.1.6): there is a Mn-catalase, and two different heme catalases: bifunctional catalase-peroxidase and a monofunctional catalase. The Mn-catalase is a very old enzyme: it appeared approximately 3.0 Ga ago [12]. It is a homo-hexameric enzyme widely distributed in bacteria and archaea [13]. The bifunctional catalase-peroxidase is a homodimeric peroxidase that also has substantial catalase activity and is only present in bacteria and fungi [5,6,8]. The monofunctional catalase is a homo-tetrameric enzyme that has mainly catalase activity [14] and is present in almost all organisms, except for some parasitic organisms [15].

Monofunctional or “typical” heme catalases have been studied for over 120 years [16] and we still have an incomplete picture of this relatively large protein that executes an apparently simple reaction at a very high rate. Monofunctional heme catalases, called from here on just catalases, are monophyletic. However, due to frequent horizontal transfers, catalases do not follow the 16S rRNA phylogeny [17,18,19]. Catalases group into three clades: clade-1 and clade-3 are SSCs and clade-2 are LSCs [17]. Clade-1 SSCs probably appeared first during evolution, then the LSCs, and finally the clade-3 SSCs [20]. Clade-1 SSCs are present in bacteria, green algae, and plants. LSCs are similar to SSCs but have an additional TD of 150–190 amino acid residues; LSCs are present only in bacteria and filamentous fungi. Clade-3 SSCs are in most phyla but not in Viridiplantae and have the particularity to bind NADPH [21].

In contrast to most animals that maintain a relatively constant internal medium, micro-organisms are exposed to sudden and large variations in their environment: different nutrient availability, low or high O_2_ concentration, low or high-water content, presence of competitors that produce H_2_O_2_, etc. Environmental conditioning is reflected in the number of catalases that these organisms have: micro-organisms and plants have a whole battery of catalases, while most animal cells have only one, mainly peroxisomal catalase. Many bacteria have a catalase-peroxidase, a LSC, and a Mn-catalase [20]. Filamentous fungi generally have one or two catalase-peroxidases [6], two LSCs, and one to seven clade-3 SSCs [19]. Plants have one to eight clade-1 SSCs [22,23].

## 2. Catalase Expression in Different Organisms

Catalases are abundant and widely distributed in most bacterial phyla except Chlorobi [20]. In *Escherichia coli*, the catalase-peroxidase HPI (encoded by the *katG* gene) is the main catalase activity during growth; the LSC HPII (encoded by *katE*) is induced during the stationary growth-phase and, together with a Mn-catalase is related to stress resistance [24]. During growth, *Bacillus subtilis* expresses an H_2_O_2_ inducible clade-3 SSC (encoded by *katA*) and during the stationary phase and other stress conditions a LSC (encoded by *katE*). In spores, there is catalase activity in the spore coat layer (KatX), probably the Mn-catalase (encoded by *ydbD*), which is important for spore germination [25,26].

Saccharomycetes have only a cytosolic and a peroxisomal SSC, while filamentous ascomycetes have one to several SSCs, which generally do not have a PST1 signal, and most of them are probably not peroxisomal [19]. Furthermore, filamentous ascomycetes have two LSCs: an L2-type, which has a signal peptide for secretion, is associated to the cell wall, and is usually induced by stress and the start of cell differentiation, and an L1–type, which is cytosolic and associated with non-growing cells and spores [27,28]. *Neurospora crassa* LSCs catalase-1 (CAT-1) (L1) and catalase-3 (CAT-3) (L2) are the main catalase activities during the asexual life cycle; CAT-2, a catalase-peroxidase, is also present but has low catalase activity compared to the two LSCs. CAT-1 and CAT-3 are expressed differentially: CAT-1 is highly accumulated in asexual spores (conidia), constituting 0.6% of total protein [11]; CAT-3 is present in the cell wall of conidia as a minor activity. During germination of conidia there is oxidative stress, singlet oxygen is produced [29], and CAT-3 becomes oxidized during the first 10 min of conidia germination [28]. CAT-1 represents the main catalase activity during germination and the start of exponential growth. It then becomes diluted by growth, and CAT-3 is increasingly expressed and accounts for the main catalase activity henceforth [28]. CAT-3 is induced under stress conditions, such as exogenous H_2_O_2_, Paraquat, cadmium, uric acid, nitrate, or by heat shock [28]. CAT-1 is induced at the late stationary phase and late during conidiation [28].

The process of asexual spore formation in *N. crassa* starts by adhesion of air exposed hyphae. From the adhered hyphae, aerial hyphae grow and ramify, and thereafter conidia are formed at their tips [30]. Asexual development is elicited by oxidative stress: production of reactive oxygen species increases, which causes a NAD(P)H and glutathione redox imbalance, protein oxidation and degradation, and rapid changes in enzyme activities [31,32,33]. CAT-3 is induced during hyphal adhesion and development of aerial hyphae, and CAT-1 is expressed when conidia are formed [28]. In CAT-3 null mutant strains there is precocious conidiation with increased amounts of aerial hyphae and conidia [32,34].

Plants have several clade-1 SSCs, which are peroxisomal enzymes but seem to have different functions: In *Arabidopsis thaliana* and rice, a leaf catalase is necessary for growth under conditions where photorespiration is relatively active and shows a peak early in the light period; another enzyme is related to β-oxidation and shows particularly high expression in male flower parts (stamen (anther and filament) and pollen), and a third catalase is associated with senescent leaves [22,23].

Remarkably, the *Caenorhabditis elegans* genome encodes tree catalase genes in tandem: one for a peroxisomal enzyme, one for a cytosolic catalase, and a third not characterized. Lack of peroxisomal catalase causes a progeroid phenotype in *C. elegans* and exhibits a decreased egg laying capacity [35]. In Dauer larvae, a developmental arrested stage during which worms do not feed, the level of the cytosolic activity is increased, whereas the level of the peroxisomal activity is similar to that found in active nematodes [36]. Absence of the cytosolic enzyme reduces the adult lifespan in wild-type animals and eliminates the extension of the adult lifespan in other mutant strains [12].

Interestingly, the free living trypanosomatids have a cytosolic catalase but the blood-dwelling parasites *Trypanosoma cruzi* and *Trypanosoma brucei* lack catalase; the expression of a heterologous catalase in *T. brucei* affects the growth potential of trypanosomes in vivo in both the insect and the mammalian hosts, probably indicating that blood-dwelling parasites, in the low O_2_ environment in which they live, require a certain level of H_2_O_2_ which is deregulated in the presence of catalase [15].

In mammalian cells, catalase is usually an abundant peroxisomal enzyme. However, during oxidative stress, part of the enzyme is retained in the cytosol in an active form [37]. Expression of catalase is altered in cancer cells [38]. In these cells, part of the catalase is bound to the cell surface [39].

## 3. Catalase Structures and Their Active Site

**Figure 1:**
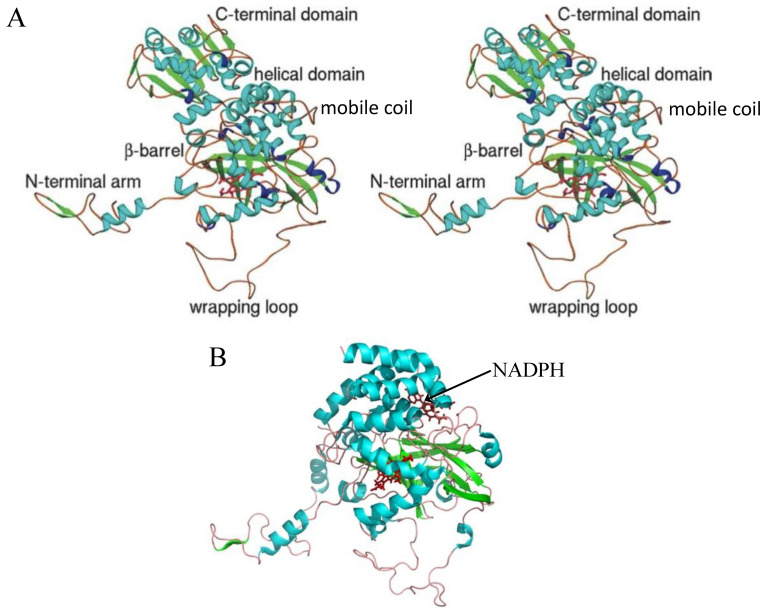
Structural regions in the monomer of CAT-1 and beef liver catalase (BLC). (**A**) Stereo view of a CAT-1 monomer with the heme in red, β-sheets in green, α-helices in blue, helices 3_10_ in dark blue, and coils in orange. The view is from the Q axis and the P and R axes are in plane with the page. Reprinted with permission from Figure 2A in ref. [41] 2004, Elsevier (**B**) Monomer of BLC with similar colors and orientation to the CAT-1 monomer. Arrow indicates the bound NADPH.

Currently, 22 catalase structures are available in the Protein Data Bank (PDB) (Table 1): 17 SSCs (13 clade-3, four clade-1) and five LSCs (clade-2). Catalases are homo-tetramers with a 222 molecular symmetry: a dimer of two intertwining subunits. In each subunit, the following structural regions can be identified (Figure 1): the N-terminal arm (varies considerably in length; but there are 47–58 amino acids from the first conserved Thr to the essential His of the active site), the β-barrel domain (258–269 amino acids), the “wrapping domain” (104–126 amino acids), the α-helical domain (73–92 amino acids), and, in LSCs, a mobile coil (29–35 amino acids) and a TD (150–190 amino acids).

**Figure 2:**
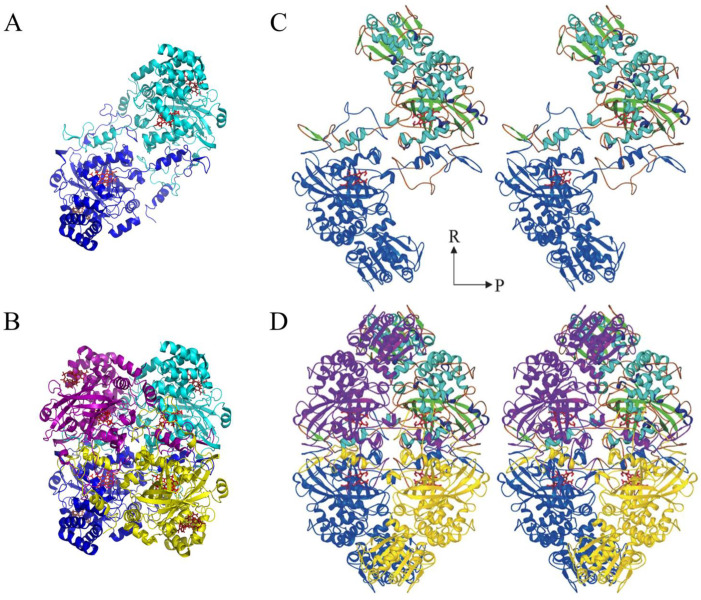
Dimer and tetramer of BLC and CAT-1. (**A**) dimer and (**B**) tetramer of BLC. (**C**) Stereo view of a CAT-1 dimer with Q related subunits. The N-terminal arm of each subunit hooks into the wrapping domain of the other subunit. (**D**) Stereo view of the CAT-1 tetramer. Figures (**C**,**D**) are reprinted with permission from ref. [41] 2004, Elsevier.

The N-terminal arm folds back, and its end hooks into the wrapping domain of the Q-axis related subunit and vice versa (the PQR nomenclature in [40] is followed) (Figure 2). The β-barrel domain is the most conserved part of the enzyme. It consists of an eight-stranded antiparallel β-barrel with six α-helical insertions in the turns between the strands. The first part of the wrapping domain contains a highly conserved α-helix (helix 9) that carries the essential Tyr, which is the proximal heme-iron ligand. The rest of the wrapping domain forms a large loop that loosely wraps around half of the β-barrel domain. The helical domain has four contiguous helices and forms part of the lateral channel where NADPH binds in clade-3 SSCs (Figure 1 and Figure 2). In LSCs, the mobile coil impairs the adenine binding of NADPH, but the “nicotinamide binding site” remains open. The TD has a conserved “flavodoxin-like” fold, although its primary sequence is poorly conserved.

**Figure 3:**
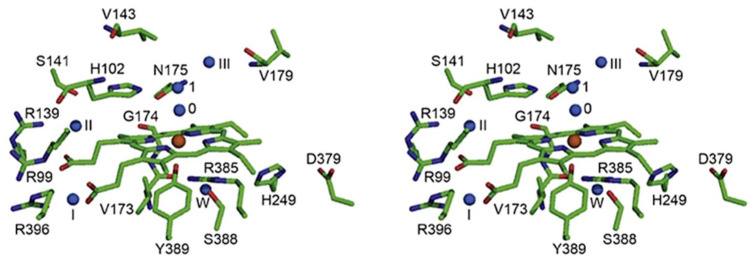
Stereo view of the CAT-3 active site. Shown are the propionic acids of the heme that make salt bridges with three conserved Arg residues and the conserved amino acid residues in the proximal and distal side of the heme. Water molecules are shown in blue. Reprinted with permission from ref. [11] 2009, Elsevier.

**Figure 4:**
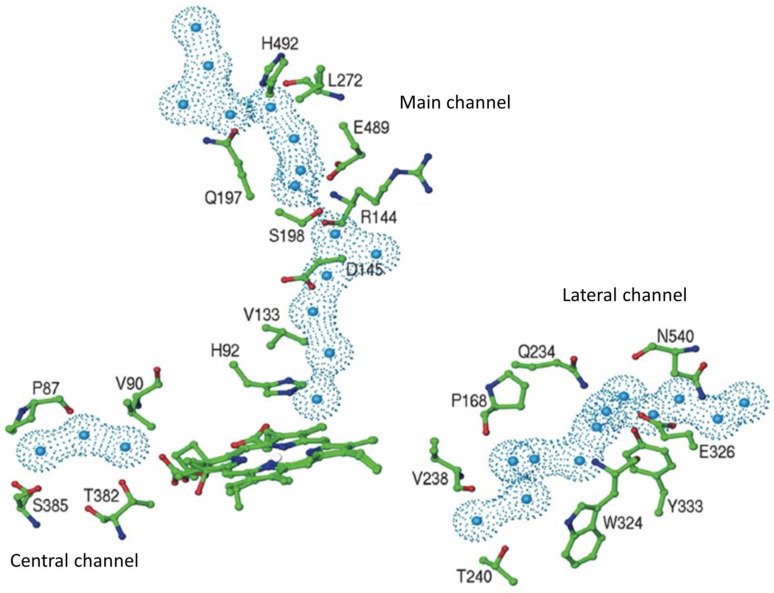
Water molecules and some of the residues lining the main channel, the lateral channel, and the central channel of CAT-1. Only the first three water molecules of the central channel are shown, the second three are already in the central cavity. Water molecules have a radius of 1.4 Å. Reprinted with permission from [41] 2004, Elsevier.

The prosthetic heme group lays in between the β-barrel, the α-helix 3, and the α-helix 9 (Figure 1). It divides the hydrophobic cavity into a distal and proximal half. In the distal heme pocket, the imidazole ring of the essential His and a conserved Phe lay parallel to the plane of the heme group (Figure 3 and Figure 4). The propionic acids of the heme make salt bridges with three conserved Arg residues (Figure 3). Close to the essential His there is the catalytic Asn and a Ser, which are the conserved amino acid residues of the active site (Figure 3). At the proximal side, the Tyr ligated to the Fe, the conserved Arg and Val residues, and an Asn or His build together a net of hydrogen bonds (Figure 3).

### 3.1. The Main Channel and the Other Conserved Channels

One of the intriguing characteristics of catalases is the different conserved tunnels these enzymes present. The main channel is very long; it goes from the protein surface near the α-helical domain to the deep-buried active site. Its entrance is funnel-shaped and hydrophilic, and the final section (FS) is narrow and hydrophobic and ends at the His of the active site. The FS is oriented almost perpendicular to the plane of the heme (Figure 4).

In SSCs, the main channel is ~30 Å long, and the entrance and the FS are continuous; in LSCs it is ~45 Å long, and the entrance ends in a small cavity (the gate) that is separated from the FS by the hydroxyl of a Ser or Thr (Figure 4). This apparent bottleneck of the canal disappears in a molecular dynamics (MD) study, particularly in the presence of H_2_O_2_ [19]. Broadening of the main channel in the presence of 30% H_2_O_2_ was also found in *Saccharomyces cerevisiae* catalase-A (SCC-A) [42]. The gate is formed by a loop of four amino acids (Q, A, Q/A, S/T) that is absent in SSCs. A cis-Pro at the N-side and Ala, His at the C-side invariably flank these four residues in LSCs. The sequence cis-P, Q, A/G, Q/A, S/T, A, H is a characteristic of the main channel in LSCs [11,41]. The gates from the P-axis related subunits are joined by the interconnecting channel which has two strings of paired water molecules [43].

**Figure 5:**
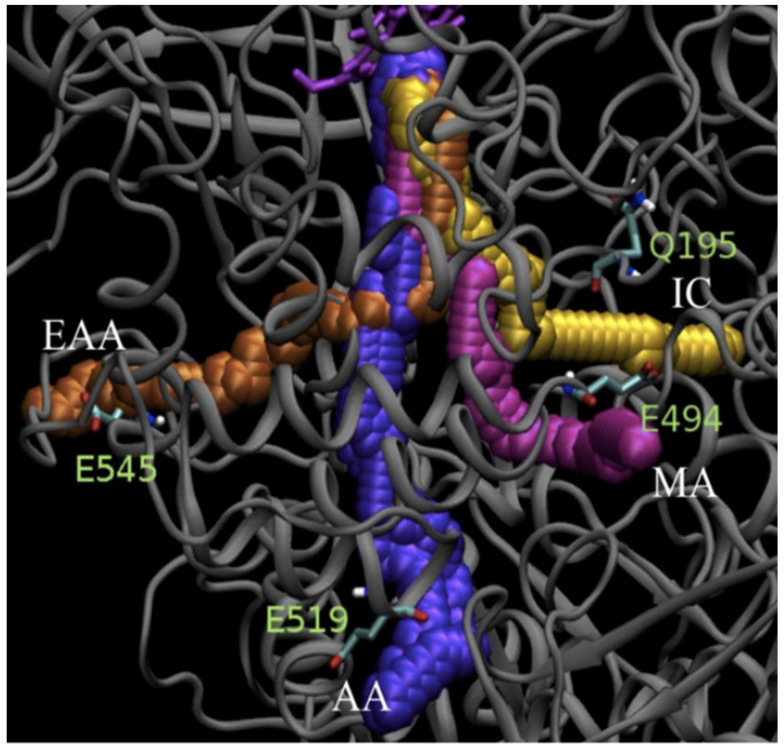
Permeability of accesses and channels in CAT-1. Access to the gate and the active site was determined with the CAVER program. The starting point was at the Fe of the heme. Traces define the main access and channel (MA) (pink), the auxiliary access (AA) (purple), the interconnecting channel (IC) (yellow) and the entrance to the auxiliary access (EAA) (orange). All these traces converge at the gate. Accesses are identified by the following amino acid residues: E494 in MA, E519 in AA, Q195 for IC, E545 for EAA. The heme is depicted in magenta. Reprinted with permission from ref. [43] 2010, Elsevier.

In both L1- and L2-type fungal LSCs there is an auxiliary access that also leads to the gate, and this tunnel is even longer than the main channel (Figure 5). The auxiliary access is shaped by the TD in both types of fungal LSCs [19,43]. Additionally, in CAT-1 there is an entrance to the auxiliary access, which is not present in CAT-3 [43] (Figure 5).

The lateral channel goes from the NADPH binding site in clade-3 SSCs to the proximal side of the active site near to the viny group of pyrrole ring II (ring I in His-IV catalases) (Figure 4) (see below). This conduit is conserved in all catalases and is not made by the interaction of two subunits as it is for the other channels. In a MD simulation, some water molecules were observed to exit the protein through the lateral channel [42].

There are other conserved channels in catalases. From the FS, a minor channel initiates but does not lead to the protein surface in HPII, PVC, CAT-1, and CAT-3. It has only two water molecules in the crystal structure of CAT-1 [41]. This channel is present in SSCs and runs from the FS to the binding site of NADPH in clade-3 catalases [44]. The central channel communicates the heme with the central cavity of the tetramer, which is the easiest way for the enzymatic products to leave the heme cavity (Figure 4). Finally, there is an interfacial channel that runs between the interfaces of the two R-axis related subunits.

The main channel carries the H_2_O_2_ to the active site: upon incubation with H_2_O_2_ of a crystal of an inactive HPII variant (H128N), H_2_O_2_ has been detected in the main channel and at the active site [45]. A SCC-A variant (V111A) that broadens the canal has increased peroxidatic activity probably because substrates can more easily reach the active site than in the wild-type enzyme [44]. MD studies confirmed that the main channel conveys H_2_O_2_ to the active site [43,46,47].

### 3.2. The Size of the Main Channel in Different Catalases

Compounds that inhibit catalase activity or react with its active site vary in size. Usually, their accessibility to the active site is higher in SSCs than in LSCs. Cyanide and hydroxylamine, which are similar in size to H_2_O_2_, can react directly at the active site and inhibit LSCs. However, cyanide reacts with HPII at a much slower rate than BLC; upon modification of heme *b* to heme *d* in HPII, cyanide binding to the active site increases [46]. CAT-1 is more sensitive to cyanide than HPII and the *Aspergillus niger* CAT-R, and the CAT-1 with an oxidized porphyrin ring (heme *d*) also has an increased cyanide sensitivity [10]. Cyanide has been detected binding linearly to the Fe of the heme of Human Erythrocyte catalase (HEC) [47].

Azide is larger than H_2_O_2_; compared to SSCs, a concentration ten to a hundred times higher is required to inhibit the LSCs [9,10]. Azide has been found to bind directly to the Fe of the heme in SCC-A [44].

3-amino-1,2,4-triazole (3AT) is a much larger compound than H_2_O_2_. It is considered a specific and irreversible inhibitor of catalases that reacts with the intermediate Cpd I. Usually, millimolar concentrations of 3AT and long incubation times (min), and low concentrations of H_2_O_2_ are necessary to obtain inhibition [48]. 3AT reacts with the protein of the BLC [48]; it binds to the His of the active site in HEC [47] and *Penicillium vitale* (now *janthinellum*) catalase (PVC) [49]. 3AT has also been detected at the nicotinamide binding site of the lateral channel in a fungal L2 LSCs, the *Mycothermus thermophilus* (*Scytalidium thermophilum*) catalase (CATPO) [50], which is very similar in sequence and structure to the *N. crassa* CAT-3. Thus, 3AT is the compound of largest size that can enter the active site in LSC: it enters in one (PVC) but not in another LSCs (CATPO), although both catalases belong to the same L2-type and are very similar in sequence.

Peracetic acid, methyl-peroxide, and even *tert*-butyl-hydroperoxide might enter the active site of *Exiguobacterium oxidotolerans* (EKTA) clade-1 catalase, which has a wider FS than BLC and *Micrococcus lysodeikticus* catalase (MLC) [51]. MLC has a narrower entrance to the main channel than BLC [52], which determines a reduced access of hydroperoxides to the active site in MLC [51]. Peracetic acid, although considerably larger than H_2_O_2_, can reduce the ferric CAT-3 into Cpd I and the resulting acetate is detected in the FS [53,54], similar to PVC and *Helicobacter pylori* (HPC) [55]. In the *Proteus mirabilis* catalase (PMC) peracetic acid forms Cpd I but no acetate is observed in the main channel, suggesting that in this catalase it flows out rapidly from the main channel [56]. In PVC, peracetic acid probably also enters through the lateral channel to the proximal side of the heme [55].

### 3.3. Rules for Prediction of NADPH Binding and Heme Orientation

Clade-3 catalases bind NADPH [21,57] (Figure 1). In our 2012 review, we suggested that amino acid residues 203 and 305 (HEC 1F4J numbering) determine NADPH binding: an Arg in position 203 is required for the interaction with the phosphate groups of NADPH and a His or Gln in position 305 allows for binding of NADPH, whereas acidic residues and bulky residues in one of these sites precludes NADPH binding [19]. This holds for the newly determined structures of both clade-3 and clade-1 catalases (Table 1). So far, the HPC is the only clade-3 catalase that does not bind NADPH [58]. In fact, instead of Gln or His of other clade-3 catalases, HPC has a Leu in site 305, which is incompatible with NADPH binding (Table 1).

Each clade also has a specific heme orientation: clade-1 and clade-2 have the active site over pyrrole ring IV (His-IV), while clade-3 catalases have the active site over pyrrole ring III (His-III). A bulky hydrophobic amino acid residue (Val or Ile) in position 217 (HEC 1F4J numbering) is associated with His-IV, whereas a small amino acid residue (Gly or Ser) is associated with His-III [3]. This rule holds for the newly determined structures of all clades (Table 1).

Table 1 Reported crystal structure of catalases at the PDB and their characteristics. SSC or LSC, clade and heme type are indicated. Prediction of heme orientation: active site over porphyrin ring III or IV is related to the presence of a small or a bulky amino acid residue at site 217, respectively (HEC 1F4J numbering). The capability of binding NADPH is related to an Arg or a positively charged amino acid residue at site 203 and a Gln or His at site 305. An acidic or bulky residue in one of these two sites precludes NADPH binding. So far, HPC is the only clade-3 catalase that does not bind NADPH because it has a Leu at site 305 instead of Gln or His.

The HEC Val217 corresponding residue in HPII is Ile274. When the Ile274 is substituted by the less bulky hydrophobic residue Val or Ala, both heme orientations were obtained in equal proportion in I274V and a predominant His-III orientation in I274A, indicating that the bulkiness of this residue determines the His-IV orientation. Two other variants (I274G and I274C) are more difficult to interpret due to the peculiar features they present [59]. In the fungal L2 LSC CATPO, similar enzyme variants were obtained in the equivalent residue (Val228), but the heme orientations were not analyzed [60].

Unfortunately, there are no structures reported for the very conserved fungal SSCs group F2 [19] that have a Lys or Arg in the 305 site and a His in position 207, which in principle would allow for NADPH binding and have a hydrophobic residue (C) in position 217, which would predict an active site over pyrrole ring IV [19].

## 4. Catalase Reaction Mechanism and Kinetics

**Figure 6:**
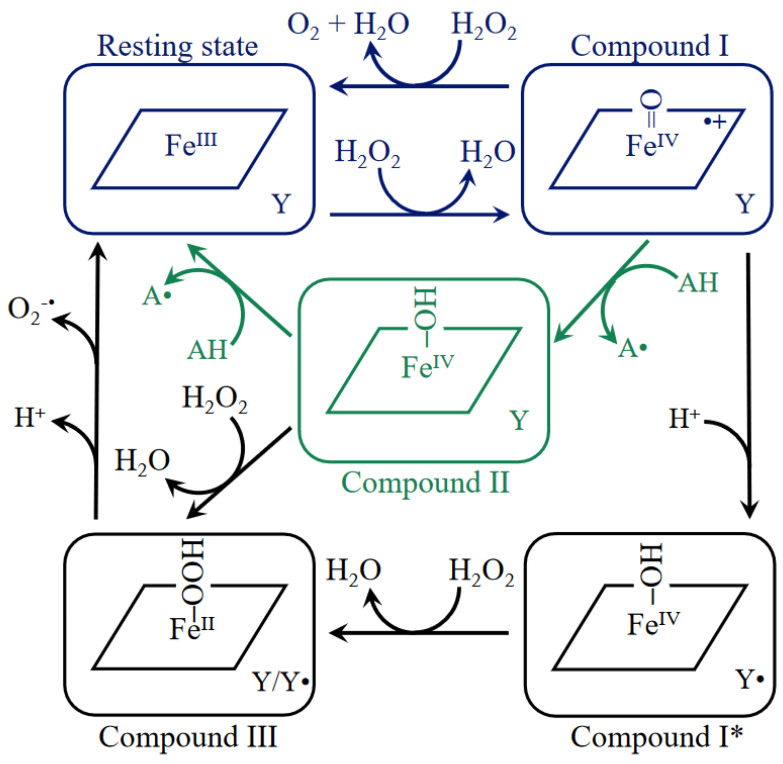
Scheme of catalase reactions. Shown in blue are the two steps of the catalase reaction which take place at a high rate; in green, the peroxidatic reaction which occurs at a much slower rate with reducing substrates (AH) at a much higher concentration than H_2_O_2_; in black, the formation of intermediates that inhibit the catalatic reaction. The parallelogram denotes the porphyrin ring and Y a Tyr in the protein that can form a radical, Y^•^.

Catalases disproportionate H_2_O_2_ into O_2_ and 2 H_2_O. Dismutation of H_2_O_2_ takes place in two-steps: the enzyme in its high spin Fe^III^ state gives away two electrons to a molecule of H_2_O_2_, one electron from the iron and one from the porphyrin ring, forming a water molecule and the oxoferryl π-cationic porphyrin radical (**^•+^**Por–Fe^IV^=O) known as Cpd I [61]. Then, a second H_2_O_2_ molecule is oxidized, giving two electrons to Cpd I to produce one dioxygen and one water molecule and the enzyme in its initial Fe^III^ state (reactions 1 and 2) (Figure 6).

Enz (Por–Fe^III^) + H_2_O_2_ → Cpd I (^•+^Por–Fe^IV^=O) + H_2_O (1)

Cpd I (^•+^Por–Fe^IV^=O) + H_2_O_2_ → Enz (Por–Fe^III^) + H_2_O + O_2_ (2)

Cpd I is a reactive intermediate and can subtract an electron from a reducing agent, forming a hydroxoferryl-porphyrin (Por–Fe^IV^–OH) known as Compound II (Cpd II) (reaction 3) or can remove an electron from a vicinal residue forming an amino acid radical, usually a Tyr or Trp radical [62], and the hydroxoferryl-porphyrin at the active site (Por–Fe^IV^–OH) + ^•^Tyr, termed compound I* (Cpd I*) (reaction 4) (Figure 6):

Cpd I (^•+^Por–Fe^IV^=O) + e^−^ + H^+^ → Cpd II (Por–Fe^IV^–OH) (3)

Cpd I (^•+^Por–Fe^IV^=O) + Tyr + H^+^ → Cpd I* (Por–Fe^IV^–OH) + ^•^Tyr (4)

Tyr radicals have been detected in BLC [62] and HEC [47] and a theoretical analysis indicated that a proton transfer determines a Tyr radical formation in HEC but not in PVC [63].

When there is a high H_2_O_2_ concentration, both Cpd II and Cpd I* can react with H_2_O_2_ to form a hydroperoxoferrous-porphyrin (Por–Fe^II^–OOH) compound III (Cpd III) (reactions 5 and 6) (Figure 6):

Cpd II (Por–Fe^IV^–OH) + H_2_O_2_ → Cpd III (Por–Fe^II^–OOH) + H_2_O (5)

Cpd I* (Por–Fe^IV^–OH) + ^•^Tyr + H_2_O_2_ → Cpd III (Por–Fe^II^–OOH) + ^•^Tyr + H_2_O (6)

Cpd I*, Cpd II, and Cpd III are inactive for the “catalatic” activity; accumulation of Cpd III with increasing concentration of H_2_O_2_ inhibits SSCs. In BLC, the Cpd III slowly decays to the ferricatalase [64].

The LSC CAT-R forms Cpd II and Cpd III with a low flux of H_2_O_2_ and long incubation times; however, due to a high rate of conversion of Cpd III into ferricatalase and to a low rate of conversion of Cpd I to Cpd II usually no catalatic inhibition is observed [64].

CAT-1 [10] and CAT-3 [11] are actually activated by H_2_O_2_: H_2_O_2_ saturation kinetics present two components, one between 1 and 200 mM H_2_O_2_ and another between 100 mM and molar concentrations of H_2_O_2_. The first component tends to be saturated at 200 mM and the second in the molar range, but both never reach saturation. Thus, both types of catalases do not follow Michaelis–Menten kinetics: SSCs because of substrate inhibition and LSCs due to substrate activation. Thus, without performing a detailed saturation kinetic experiment, the apparent *K*_M_ and V_max_ reported for various catalases are meaningless.

Using a quantum mechanical approach in conjunction with ab initio MD of the active site, the reaction mechanism for the second step of the catalase reaction in the PVC and HPC has been simulated [65]: the reactants state complex (Cpd I–H_2_O_2_) evolved to another intermediate (Cpd II-like) involving the transfer of a hydrogen atom, an electron to the porphyrin cation radical and a proton to the oxoferryl. Then, the distal His as an acid-base catalyst mediates the transfer of a proton followed by an electron transfer to the iron to form a water molecule at the Fe^III^ site and the O_2_ close to the distal Asn.

In HPC, a second pathway can take place: instead of the His mediated proton transfer, a hydrogen atom is transferred from the peroxyl radical. Irrespective of the pathway, the reaction proceeds by two one electron transfer steps, formally described as H^•^/ET-H^+^ or H^•^/H^•^. The two pathways are accompanied by the motion of the active site histidine: which moves toward the heme when it transfers a proton to the hydroxoferryl or moves away from the heme when the peroxyl radical transfers a hydrogen atom. The steps with the highest energy barrier along each pathway correspond to changes in the hydrogen bond pattern.

Until now, a brief review of the structure and activity of catalases has been given. However, to better understand how monofunctional heme-catalases work, there are several questions that need to be addressed. Here, we will discuss the following issues: (1) How do catalases select H_2_O_2_ in a sea of water? (2) How do catalases deal with an unproductive reactive Cpd I intermediate? (3) What is the origin of the heme *b* to heme *d* modification? and (4) What is the origin and function of the TD in LSCs?

## 5. How Do Catalases Select H_2_O_2_ in a Sea of Water?

A most informative MD study on how H_2_O_2_ reaches the active site was done with CAT-1 [43,66]: The whole tetrameric enzyme was simulated in both a box of water molecules and 6 M H_2_O_2_. H_2_O_2_ molecules were placed outside the protein. After reaching equilibrium, root-mean-square fluctuation followed closely the B–values of the crystal structure; however, amino acid mobility was generally larger in water and even more in H_2_O_2_.

Channel permeability to the solvent was tested with the program CAVER. Starting at the Fe of the heme, tracks that were at least the size of a water molecule were analyzed. The main and auxiliary accesses, the entrance to the auxiliary access, and the interconnecting channel all converge at the gate and are permeable to the solvent (Figure 5). In contrast, no traces are detected for the central, the minor, the lateral, and the interfacial channel [43].

**Figure 7:**
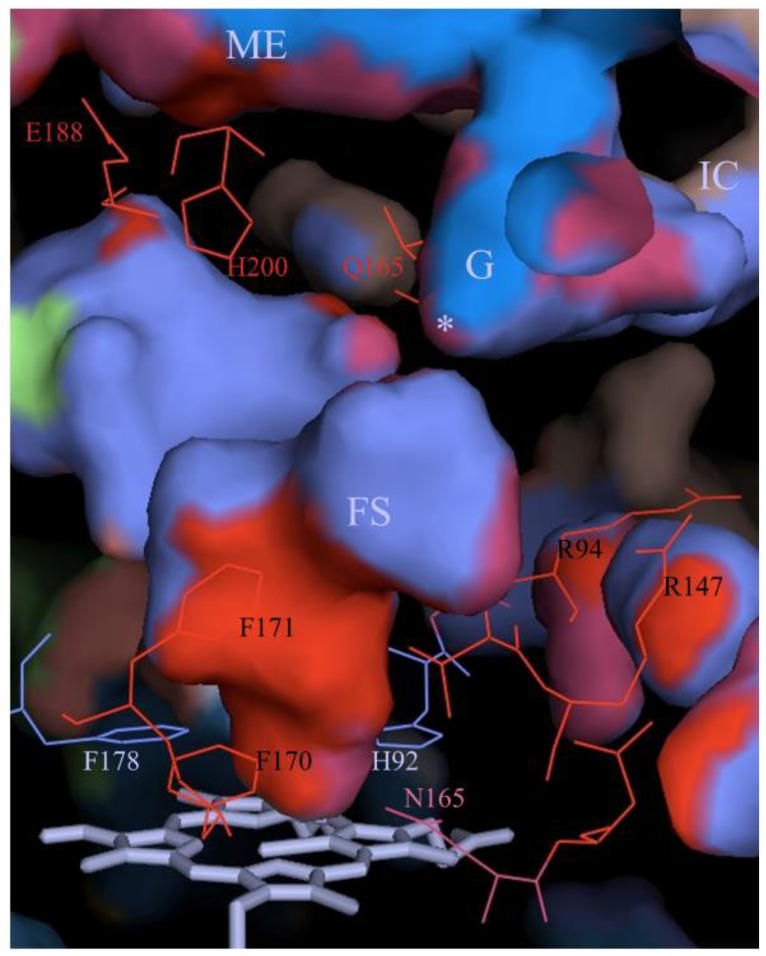
Coordinated amino acid movement in the main channel of CAT-1. Shown are the internal surfaces of the main channel of the main entrance (ME), the gate (G), the asterisk indicating the interruption between the gate and the FS by Ser198, the final section (FS), and the active site at His92 and Asn165. Marked in red and pink lines are the amino acid residues that present coordinated movement (red indicates the most coordinated). Shown in light blue lines are the less mobile amino acid residues. The heme is depicted with gray sticks. The interconnecting channel (IC) connects the gates from the P-axis related subunits.

The B-values of the gate are low in the crystal structure, while the mobility of the gate loop residues is high in water and in H_2_O_2_. The bottleneck in the crystal structure between the gate and the FS disappears in the simulation with water, and the region 12–22 Å from the Fe of the heme is considerably widened in the simulation with H_2_O_2_ [43]. Other amino acid residues that have increased mobility in water and in H_2_O_2_ are located in different channels [66]. A high mobility in H_2_O_2_ is related to amino acid residues localized at the main access, the auxiliary access, the entrance to the auxiliary access, the gate, the FS, the interconnecting channel, the minor channel, and the interfacial channel. In contrast, the His92 and Phe178, which have strong van der Waals interactions with the heme, show very low mobility (Figure 7).

Most interesting is the fact that the movement of certain amino acid residues is coordinated in H_2_O_2_ [66] (Figure 7). It is notable that the inflow of both water and H_2_O_2_ into the FS is mainly through the gate and there is no passage of solvent molecules from the FS to the gate. The exit of solvent molecules occurs at a site in the FS. Pronounced and coordinated motion from the channel entrances to the gate could allow for a rapid flow of H_2_O_2_ into the FS and the active site.

Since water is always in great excess over H_2_O_2_, how do catalases contend with the competing effect of water? Already, after a 60 ns simulation, there is proportionally more H_2_O_2_ molecules in the first 5 nm around the protein (1 H_2_O_2_/6 solvent molecules) than in the bulk solvent (1 H_2_O_2_/9 solvent molecules) [43]. This is due to the prevalence and distribution of amino acid residues that have an increased residency for H_2_O_2_. The residues residence time variance of H_2_O_2_ compared with water molecules has the following sequence in decreasing order: K > R = W > C = H > M > P = E = D > F > Y; residence time is 3.5–fold higher in Trp and Arg compared to Tyr. There is a preponderance of His, Pro, Trp, Met, and Cys and charged amino acid residues in the accesses of CAT-1. These residues are clustered, forming paths to the gate that are delimited by hydrophobic residues. The H_2_O_2_/H_2_O ratio increases in the main access and later in the auxiliary access in each subunit. By contrast, in the central cavity, after 90 ns, the H_2_O_2_/H_2_O ratio increased only to the value of the bulk phase. In the FS, the H_2_O_2_/H_2_O ratio increases even more (1 H_2_O_2_/3–4 solvent molecules) but at a different rate in each subunit and with variations in time: at the beginning of the simulation, only one subunit has an increased content of H_2_O_2_ molecules, after 50 ns three subunits, and after 80 all four FS had an increased content of H_2_O_2_. At the end of the simulation, two had a high content (1 H_2_O_2_/2–3 solvent molecules) and the other two had low content of H_2_O_2_ (1 H_2_O_2_/9 solvent molecules). In the FS, the turnover rate of water molecules is five times faster than for H_2_O_2_ [43].

The maximal occupancy sites of water in the FS coincide with the position of water molecules in the crystal structure [41]. Both water and H_2_O_2_ have a maximal residency at 4.5 (W1), 11.5 (W3), 14 (W4), and 15.5 Å (W5) from the Fe of the heme, but only water molecules occupy sites at 2.5 and 8.5 Å (W2). The absence of H_2_O_2_ at the 8.5 Å site makes improbable the “molecular ruler” hypothesis proposed for HEC, which states that H_2_O_2_, by virtue of its length would better satisfy the hydrogen bonding in the hydrophobic FS, specifically in the positions of W1/W2 and W3/W4 [47]. Higher residency values are observed for H_2_O_2_ at sites 4.5, 11.5, and 15.5 Å and less defined at 14 Å. When the W1 at the active site is displaced by H_2_O_2_ it occupies the 2.5 Å site, where H_2_O_2_ never enters [43].

**Figure 8:**
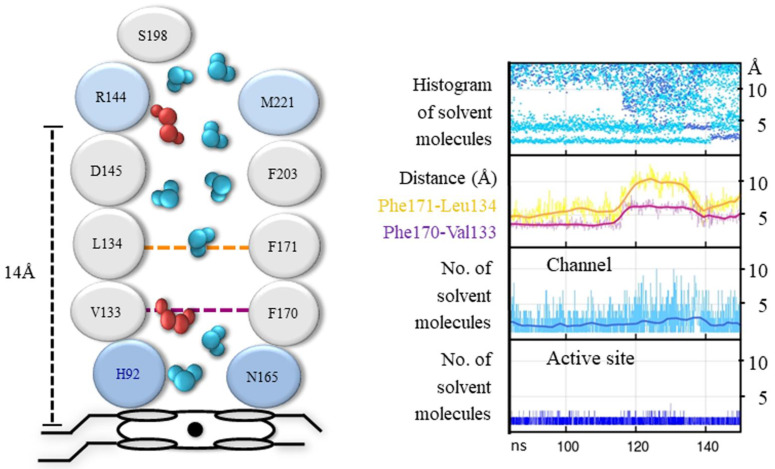
Detection of a gate valve mechanism at the FS of the main channel in CAT-1. Left: shown is a scheme of the FS, from the Fe of the heme to the gate (Ser198), a distance of ~17 Å. The circles represent the amino acid residues that line the FS, which is filled with water (blue) and H_2_O_2_ (red) molecules. Phe171 and Phe170 are very mobile residues opposite to Leu134 and Val133, respectively. Shown in blue are the R144/M221 low affinity binding site for H_2_O_2_ and the H192/N165 high affinity active site. Right: measurements from MD simulations [66], reprinted with permission a modified Figure 6D from ref. [47] 2014, Wiley). From top to bottom: the presence of solvent molecules along the 14 Å length channel (water = light blue, H_2_O_2_ = dark blue); the distance between the Phe171 and Leu134 (yellow) and the Phe170 and Val133 (magenta); the number of solvent molecules in the FS; and the number of solvent molecules at the active site His92/Asn165. Shown as a time lapse for the MD simulation (~65 ns) in which at the beginning both phenylalanine residues are very close to their opposite amino acid residues, closing the FS and thus no solvent molecules were detected in a region of ~6 Å in the FS. Then, both Phe residues are seen to rotate simultaneously, away from their opposite residues, thereby opening the FS and allowing the entrance of solvent molecules into the empty gap. A mean of five solvent molecules enters but only three reach the active site. The water molecule at the His92 is substituted by H_2_O_2_.

**Figure 9:**
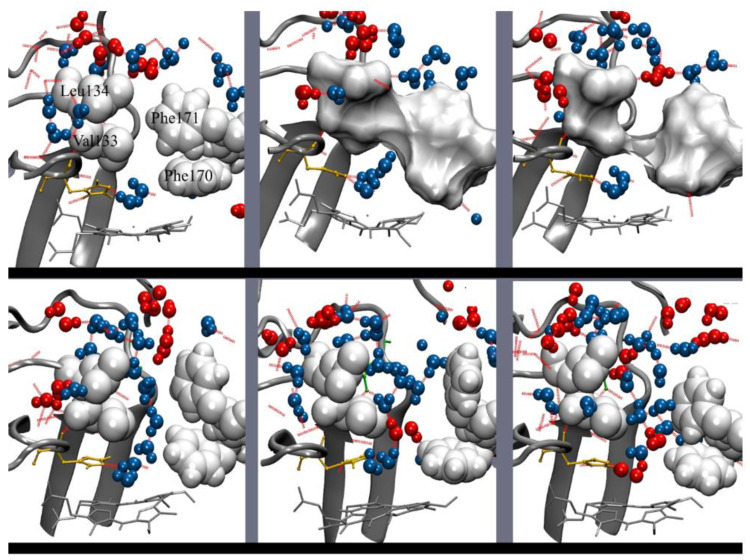
A gate valve mechanism at the FS in the main channel of CAT-1. Representation of the gate valve mechanism of CAT-1 during FS closing and opening obtained from a MD simulation. Upper panels: Phe171 rotates toward Leu134 and Phe170 toward Val133, closing the FS of the main channel, and draining solvent molecules from the FS. Water molecules (blue) are bound to the His92. Lower panels: Phe171 and Phe170 rotate away from Leu134 and Val133, respectively, allowing solvent molecules to enter the active site. The water molecule bound to His92 is displaced by H_2_O_2_ (red).

Between the gate and the active site there is a steep gradient in the number of solvent molecules: many are present at the gate, few at the FS, and very few at the active site, indicating that there is no free diffusion of solvent molecules in this section. This is due to the operation of a gate valve mechanism in the FS at the region between 5 Å and 11 Å from the Fe [66]: solvent molecules (W2–W3) are drained from this region whenever Phe170, just above the active site, and Phe171, about the middle of the FS, move rotating either toward the active site, blocking the channel, or away from it, leaving the passage open (Figure 8). Most of the time, this region of the FS is empty of solvent molecules. The gate valve mechanism operates both in H_2_O_2_ and water but the movement of these highly conserved Phe170 and Phe171 is more pronounced and coordinated in H_2_O_2_ than in water. In H_2_O_2_, both phenylalanine residues are in an open position only 35% of the time. This value is in accordance with the average number of H_2_O_2_ molecules that reach the active site, which is 1.2 per tetramer in HEC and 1.6 in MLC, even in the presence of a thousand-fold excess of H_2_O_2_ [67]. When the gate valve is open, a mean of five solvent molecules flows into the empty region, and an average of three reach the active site where they are retained (Figure 8). Usually, one of the three solvent molecules is H_2_O_2_ which substitutes the water molecule (W1) at the active site (Figure 9).

A lack of water molecules in the region of W2–W3 sites is observed in the FS of the crystal structures of CAT-3 [11] and some HPII variants [45], most probably due to the movement of these phenylalanine residues. In a MD study of PMC, most of the H_2_O_2_ molecules that were put at the gate did not enter the active site and were retained at Phe132 [68], the equivalent of Phe170 in CAT-1. On the few occasions that H_2_O_2_ is passed to the active site, the Phe132 is rotated to an open position, indicating that the gate valve system is also operative in PCM.

The movement of the two conserved Phe residues is probably critical for enzymatic activity: The replacement in SCC-A of the CAT-1 Phe170 equivalent Phe148 by valine has only 10% of the catalatic activity and no peroxidase activity and exhibits severe deficiency in heme binding and reduced stability; replacement of the CAT-1 Phe171 equivalent Phe149 by valine decreased the catalatic and the peroxidatic activity considerably [68].

The FS is hydrophobic in all catalases. In CAT-1 it is made of 14 hydrophobic amino acid residues; only the conserved Arg144 and Asp145 at the entrance of the FS and the active site are hydrophilic. To reach the active site, solvent molecules have to cross a hydrophobic region between Asp145 and His92, a distance of ~7 Å. Being longer and forming more and stronger hydrogen bonds, H_2_O_2_ can cross the hydrophobic barrier and reach the active site more easily than water molecules [66]. Furthermore, with H_2_O_2_ having a higher polarity than water, it would be better steered toward the active site by the electric potential between Asp145 and His92 [69], particularly when Cpd I is formed, which has a positive charge.

One can envisage the whole FS as the active site in catalases. It would consist of a low affinity binding site for H_2_O_2_ at Arg144/Met221, a high affinity binding site for H_2_O_2_ at His92/Asn165 and the hydrophobic region in between with the two mobile Phe residues, draining water out of the FS and regulating the passage of H_2_O_2_ from the low affinity binding site to the high affinity binding site. Having a longer residence time, H_2_O_2_ will displace water at both sites.

The function of the gate cavity would be to provide H_2_O_2_ to the low affinity binding site. This is done efficiently by selecting H_2_O_2_ over water along the paths to the gate together with a coordinated motion of certain residues from the protein surface to the gate. Because the gates of the P-axis related subunits are communicated by the interconnecting channel, H_2_O_2_ from both subunits will be channeled to one or the other FS according to the position of the gate valve system in the two subunits. Accordingly, amino acid residues of the interconnecting channels are visited by both entering and leaving H_2_O_2_ and water molecules [66]. Thus, the P-axis related subunits work together. This would explain the functional asymmetry observed in some catalases [47]. Most of the time there will be only one of the two active sites in an open position. Thus, H_2_O_2_ and water will flow from the subunit with the FS closed to the one having the FS in an open position, and this would alternate continuously. Two subunits ministering H_2_O_2_ to only one active site would reduce the formation of an unproductive Cpd I and thus the formation of Cpd I*, Cpd II and III.

Two outlets for water are required, one at the entrance of FS, probably the minor channel, which would drain water molecules from the FS due to the motion of the two Phe residues. Water molecules WA and WB, in a row with W3, at 2.6 Å from each other indicates an outlet from the FS between Phe171 and Phe203 [43]. W3, WA, and WB would be driven out by the movement of Phe171, and W2 would be pushed to the W3 site by the movement of Phe170. The second outlet is for the water molecules that reach the H92/N165 site and the water produced by the catalase reaction. From the active site, water molecules will go to the 2.5 Å site and from there out of the enzyme through the central or the lateral channel. The Val90 at the entrance of the central channel is visited only by outgoing water molecules [66], indicating that at least part of the water goes out through the central channel.

The MD results were in agreement with the complex saturation kinetics observed in CAT-1 [10] and CAT-3 [11]. Considering the whole FS as the active site of catalases, would account for the observed non-hyperbolic kinetics, cooperativity, and two active sites differing in >10-times in substrate affinity [10]. Activation by H_2_O_2_ is explained by the decreasing effect of competing water with an increasing H_2_O_2_ concentration. The gate valve system, by regulating H_2_O_2_ accessibility to the active site with H_2_O_2_ concentration, would account for the observed cooperativity, backed up by the increase with H_2_O_2_ concentration in the mobility of certain amino acid residues along the paths to the gate. The existence of two active sites differing in substrate affinity is accounted for the two binding sites of H_2_O_2_, one at Arg144/Met221, which has a low affinity, and the His92/Asn165 site, which has a high affinity for H_2_O_2_ (a much higher retention time than water) (Figure 8). H_2_O_2_ at the low affinity binding site is required to access to the high affinity site. Both sites would never attain saturation because of competing water molecules which are always in excess.

## 6. How Do Catalases Deal with an Unproductive Reactive Cpd I Intermediate?

When there is a low concentration of H_2_O_2_, which is usually the case in cells unless they are under oxidative stress, SSCs can contend with an unproductive Cpd I in different manners. A peroxidatic compound, like ethanol [70], formic acid [56,58], or nitrite [71], can reduce Cpd I to the ferric enzyme in a two-electron donation reaction. These compounds are usually present in all cells. To function as substrates, they have to be present at a concentration that is several orders of magnitude higher than that of H_2_O_2_, which is usually in the nanomolar range. Ethanol is not only produced by *Saccharomyces* and other micro-organisms but also by multicellular organisms, particularly under hypoxic conditions. Catabolism of some amino acids, mainly Gly and Ser in mitochondria, and the synthesis of sterols at the ER produce formate. Nitrite is formed via the oxidation of nitric oxide (^•^NO) or the reduction of nitrate. LSCs, probably because of their longer main channel than SSCs and the gate to the FS, have a hundred times less peroxidatic activity with ethanol and formic acid than SSCs [72].

Catalase can also use one-electron donors to reduce Cpd I to the ferric enzyme. Although both O_2_^•−^ and ^•^NO bind to the ferric catalase, inhibiting its catalatic reaction, both can react with Cpd I, forming Cpd II and then reduce Cpd II to the ferric enzyme, forming O_2_ and nitrite, respectively [73,74,75,76]. Nitric oxide has been found at the active site of BLC [77] and of *Corynebacterium glutamicum* catalase (*kat*A) [78]. Compared to other hemeproteins, BLC has a relatively high ^•^NO dissociation rate of 1.5 s^−1^ [77]. Other one-electron reducing agents, such as ascorbate, do not enter the FS of catalases but bind instead at the NADPH binding site [79].

At the protein surface, the entrance to the lateral channel in clade-3 catalases is the binding site for NADP(H). Some catalases, such as BLC, bind NADP(H) tightly in all four subunits (Figure 2) and binding affinities of dinucleotides follow the order: NADPH > NADH > NADP^+^ > NAD^+^ [21,80]. In other catalases, such as HEC and PMC, the dinucleotide binding occurs more loosely or not in all subunits [47,81]. The NAD(P)(H) binds in a folded right-handed helix conformation at two contiguous clefts in the protein: one for the adenosine and another for the nicotinamide [57]. When there is a low rate of H_2_O_2_ generation, NADPH prevents the accumulation of Cpd II. NADPH is oxidized at a slow rate in this process [82] but at three times the rate of Cpd II formation; additionally, NADPH does not increase the decay of Cpd II [83].

The bound NADP^+^ is directly reduced by external unbound NADPH; thus, there is no need for an exchange of reduced and oxidized nucleotides [80]. It has been proposed that NADPH gives two electrons to Cpd I [82] or to Cpd I* [83,84] to form the ferric enzyme. Electron transfer reaction from NADPH to Cpd I or Cpd I* could proceed by two consecutive one-electron transfers instead of one two-electron transfer [85]. A two one-electron transfer reaction agrees with the Tyr radical observed when a Phe, localized in the path from the nicotinamide binding site to the active site, is substituted by Tyr [86]. However, an interesting and convincing mechanism has been proposed that is in agreement with kinetic data and with one step of two electron transfer: a hydroxyl anion from a water molecule undergoes a reversible nucleophilic addition to the terminal carbon of the 4-vinyl group of Cpd I, producing a neutral porphyrin π-radical ferryloxo (HO–^•^Por–Fe^IV^=O) species called Cpd II′ [87]. This intermediate has reduced reactivity and thus avoids the formation of a protein radical. Formation of Cpd II′ becomes kinetically important only when the lifetime of Cpd I is increased. A hydrogen-bonded network from NADPH to the vinyl would allow two electrons to be transferred to the heme center followed or preceded by protonation, thereby restoring the catalase Fe^III^ resting state.

### Other Reactions of Catalases

The utilization of glucose oxidase or xanthine oxidase + SOD systems to produce a continuous low flux of H_2_O_2_ has been instrumental in elucidating the mechanisms of catalase reaction and the inhibition of the enzyme by its substrate [88]. However, in an in vivo system, the accumulation of an intermediate (such as Cpd II) in an interval of 20–30 min probably has little relevance in face of the fast dynamics of metabolism and its fluctuations [89] and the oscillatory behavior of enzymes, such as peroxidases [90].

Glucose oxidase is a FAD enzyme that takes electrons from glucose to reduce O_2_ into H_2_O_2_ and gluconolactone. Glucose oxidase has been used to archive electron transfer to an electrode or a compound in place of O_2_ [91]. Thus, when conditions of low concentration of O_2_ are reached, it would be important to analyze if FADH of glucose oxidase (or xanthine oxidase) can give electrons to NADP^+^, either free or bound to the catalase protein, so that the NADPH formed can transfer electrons to the active site. Transferring electrons from FADH to NADP^+^ and then to the active site would circumvent the often-raised presumption of catalase having a “good supply” of endogenous donors [92], stated much before binding of NADPH to catalases was discovered. A supply of endogenous donors from the catalase proteins seems unrealistic when many enzymatic cycles are involved.

The NADPH binding site can also bind other compounds: ascorbate binds to the adenine binding pocket in a NADPH-free BLC, reducing Cpd I into Cpd II; NADPH and other dinucleotides compete for the site and displaces ascorbate in a concentration dependent manner and according to the relative binding affinities of the dinucleotides [79].

Flavonoids (myricetin and quercetin) bind to BLC and reduce Cpd I into Cpd II under a low flux of H_2_O_2_; however, in this case, the presence of NADPH has a small effect even though the binding constant of NADPH to catalase is approximately three orders of magnitude higher than the respective constants of myricetin and quercetin [93]. The small effect of NADPH could be explained if flavonoids induce a conformational change that inhibits the binding of NADPH [94]. Alternatively, flavonoids could bind and act in another site of the protein, and NADPH bound at its site would not prevent, in this case, the Cpd II formation. One should consider that ortho-dihydroxy benzene containing flavonoids can bind to proteins nonspecifically and can auto-oxidate forming O_2_^•−^ which can reduce Cpd I into Cpd II (see below). In fact, the amount of Cpd II formation by myricetin is reduced by one third in the presence of SOD [93].

It has been described that mammalian catalases, besides their catalatic and peroxidatic activities, also have an oxidase activity. These experiments were done using 10-acetyl-3,7-dihydroxyphenoxazine (known as Amplex Red), a dye that becomes fluorescent when oxidized, which in principle, can be convenient to measure oxidase activity [95]. However, the dye is photo-oxidized, generating a resorufin radical in the presence of ambient room light or instrumental excitation light used to detect the fluorescence; the radical itself reacts with O_2_ to form O_2_^•−^ which can spontaneously dismutate to H_2_O_2_ [96]; O_2_^•−^ reacts with ferricatalase to form Cpd III [74].

This does not mean that catalase does not have an oxidase activity; clade-3 catalases actually function as NADPH oxidase when there is an unproductive Cpd I. Catechol and pyrogallol are oxidized by BLC when high concentrations (100 mM) of these compounds are used [95]. Phenols in general and salicylic acid in plant and mammalian catalases participate in one electron transfer to Cpd I and Cpd II [88,97]. The *M. thermophilus* L2 LSC CATPO is also reported to have a catechol oxidase activity. Several mutants at the nicotinamide binding site, which is also a binding site for 3AT, reduced the oxidase activity [50]. Other phenolic compounds with two hydroxyl groups in the ortho position (chlorogenic acid, (+)-catechin, and caffeic acid) are oxidized in the presence of this catalase, and the major products observed at the end of 1-h oxidation at 60 °C were mainly dimers [98].

One should be aware that catechol and other 1,2-dihydroxybenzene compounds auto-oxidize, consuming O_2_ and forming O_2_^•−^ plus quinones; quinones spontaneously dimerize [99]. Superoxide reacts with ferricatalase to form Cpd III and also can spontaneously dismutate and form H_2_O_2_ which would form Cpd I. Superoxide can reduce Cpd I into Cpd II and Cpd II into the ferric enzyme. Besides, 1,2-dihydroxybenzenes are metal chelating agents firmly binding Fe^III^ and Cu^II^ ions and thereby forming redox reactive compounds [100,101]. Moreover, catechol is capable of forming a wide range of reversible (e.g., hydrogen bonding, cation–π interaction, metal ion complexation) and irreversible cross-linking chemistries [101], for instance, with cysteines at the protein surface forming a catechol–thiol adduct. Catechol bound to catalase could give the false impression that the enzyme has a phenol oxidase activity, which is actually due to the auto-oxidizing reaction of catechol and the O_2_^•−^ produced.

If there is an intrinsic phenol oxidase activity of catalases that can be clearly separated from the auto-oxidation of 1,2-dihydroxybenzene compounds, it would be a general and probably a minor activity of catalases. If so, the name of CATPO (for Catalase-Phenol Oxidase) is hardly justified and even less CATPO as a “fourth group of catalases” (besides monofunctional heme catalases, catalase-peroxidases, and Mn-catalases) [102].

From UV-B radiated keratinocytes, previously incubated with 2′,7′-dichlorodihydro fluorescein diacetate (DCHF-DA), catalase was isolated as the source of reactive peroxides produced in excessive quantities with UV light [103]. However, DCHF is photo-oxidized in the presence of UV light giving rise to a high signal, which is mainly related to hydroxyl radical formation, particularly when the dye is bound to proteins, such as albumin and other proteins that bind Fe or have heme as a prosthetic group [104]. Catalase probably binds DCHF nonspecifically and could release Fe when irradiated with UV-B. Blue light irradiation causes partial heme destruction in sunflower catalase and oxidation of the active histidine [105] and in BLC UV (365 nm) radiation leads to the formation of catalytically inactive Cpd II and Cpd III [106]. Thus, UV radiation probably inhibits catalase activity by several mechanisms which could lead to an in vivo increase of H_2_O_2_. However, the in vitro increase in ROS is probably an artifact related to the fluorophores used to determine ROS. UV irradiation also oxidizes dihydrorhodamine 123 to generate a rhodamine 123 green fluorescence signal [107]. Both fluorophores are inappropriate for peroxide determination [108].

When a new function for catalase is suspected, it is needed and strongly recommended to do all kinds of control experiments to discard possible artifacts in the measurements.

## 7. What Is the Origin of the Heme *b* to Heme *d* Modification?

In HPII [109], PCV [110], CAT-1 [41], and CATPO [111], the pyrrole ring III of the heme is oxidized to a *cis*-hydroxyl group in C5, and a *cis*-γ-spirolactone in C6 (heme *d*). However, this should not lead to a generalized statement that all LSCs have a heme *d* instead of the heme *b* of SSCs [112] because CAT-3 does not have a heme *d* but a heme *b* [11]. In fact, it seems important to find out why CAT-3 does not oxidize its heme *b* into heme *d* since CAT-3 has a similar structure, kinetics, and physicochemical properties as CAT-1 [10,11].

For the oxidation of heme *b* into heme *d*, catalase activity is required [72,113]. To crystallize CAT-1, the enzyme was purified from the asexual spores of *N. crassa* [114], where it is highly accumulated [115]. The CAT-1 structure has a 57% occupancy of heme *b* and 43% of heme *d*, indicating that half of the enzyme extracted had not been active in the metabolically quiescent conidium [41]. We have put forward that singlet oxygen, occasionally produced catalytically or by external perturbations, would attack the double bond between C5 and C6 of ring III, forming a transient endoperoxide. Upon breaking, the endoperoxide would generate two *cis*-hydroxyl groups at these two carbon atoms. Subsequently, the propionate would form the γ-spirolactone in C6 and a water molecule [41].

In the second step of the catalase cycle, H_2_O_2_ serves as a two-electron reductant of Cpd I to form O_2_ and H_2_O. It has been proposed that two one-electron-transfer steps would lead to triplet oxygen and a pairwise movement of electrons to singlet oxygen production [115]. A qualitative analysis by means of density functional theory indicates that this scheme is not justified and that another scheme involving first proton and hydrogen atom transfer, then proton and electron transfer, is compatible with the conservation of the total spin and that triplet oxygen is the product of the reaction [116].

It is clear that singlet oxygen production at each catalytic cycle does not make biological sense since such a reactive compound would destroy the enzyme. However, the theoretical study does not demonstrate that triplet oxygen is *always* produced. The simplified model and the approximations used do not allow discarding the occasional production of singlet oxygen. Furthermore, electric fields and other external perturbations can influence the electronic properties of Cpd I [117,118], which in principle, could alter the normal catalytic cycle. Finally, the heme is excited by blue light, and O_2_ can take the excitation energy to form singlet oxygen. Thus, O_2_ at the active site could form singlet oxygen by photosensitization. If one considers that singlet oxygen is produced once every 10^8–9^ catalytic cycles or that electric or magnetic fields or photosensitization reactions can occasionally form singlet oxygen in catalases, these possibilities could explain the modification of heme *b* into heme *d*.

Singlet oxygen is a very reactive compound, and as soon it is produced, it will react with the nearest double bond it encounters. The double bond of pyrrole ring III, at the start of the central channel that leads to the central cavity in LSCs, is the nearest double bound from the catalase active site [41]. HSPII, PVC, CAT-1, and CATPO all have a permeable central channel that goes from the heme to the central cavity, which is filled with water molecules. In these LSCs, O_2_ and water would exit the protein through the central channel, which is the shortest and easiest way out of the enzyme [41]. It is also revealing that in the HPII variants that change the orientation of the heme, the pyrrole ring that is oxidatively modified is the one that is a the start of the central channel [59]. In contrast to the other four LSCs, in CAT-3, Q422 (instead of Ala or Met in the other LSCs) blocks the central channel, and the central cavity is occupied by R398 from the four subunits (G in the other LSCs) [11]. Thus, O_2_ generated at the active site of CAT-3 cannot leave through the central channel and would get out of the active site through the main or the lateral channel or even through a non-described hydrophobic path. Thus, the occasionally formed singlet oxygen in this enzyme would not react with the double bond of pyrrole ring III and would not form heme *d*.

Singlet oxygen production by heme enzymes has been documented [119,120]. Peroxidases produce singlet oxygen that can be used for biocatalytic synthesis [121]. Biliverdin and Met-sulfone formation in PMC is associated with Cpd I formation [122], and the reaction of singlet oxygen with the heme and the Met that is close to the active site might be an explanation. Singlet oxygen can not only break the heme, but it can also break a peptide chain. The HPII variant F413Y could be an example [123], because singlet oxygen preferentially reacts with certain amino acid residues, and Tyr is one of them. A great variety of heme proteins have an amino acid residue (Cys, SeCys, Met, His, Trp, Tyr) bound to one or the two vinyl groups of the heme [124]. The double bound of the vinyl group is a site for singlet oxygen reaction and so are the amino acids listed. Many other modifications in amino acid residues from the heme pocket have been described [124]. Most of these modifications occur spontaneously and could be related to the occasional formation of singlet oxygen at the active site of the heme proteins, such as the catalase-peroxidase, for which singlet oxygen activation of the Trp to form the M-W-Y adduct has been suggested [115].

CAT-1 and CAT-3 can be stored for months without significantly losing their activity. However, within a few weeks of storage in a refrigerator, a stepwise change to a more acidic isoelectric point (pI) is observed unless the enzyme is stored under argon [125]. In a refrigerator, the stored enzyme will be subjected to strong magnetic fields every time the compressor is turned on, which could transiently alter the electronic properties of the enzyme. We do not know if magnetic fields can lead to the formation of singlet oxygen at the active site of catalase. However, when a recently purified CAT-1 is exposed to a pure source of singlet oxygen that diffuses through a small gap of air to reach a drop of enzyme solution, a similar increase in electrophoretic mobility is attained within minutes; no other reactive oxygen species can bring about this pI change. The presence of a singlet oxygen quencher in the solution of CAT-1, such as His, Trp, Tyr, and 5-amino salicylic acid, prevent the change in the electrophoretic mobility [125]. Catalases from different sources, bacterial, fungal, plant, and animal, are likewise oxidized by singlet oxygen, causing a shift in electrophoretic mobility that is prevented by singlet oxygen quenchers [125]. No change in activity was observed in all these enzymes. In CAT-1 there is an increase in heme asymmetry [125], probably related to heme *d* formation but not in CAT-3 [11]. Both, non-oxidized and oxidized CAT-1 have similar kinetics, small differences in stability under extreme conditions, and a higher sensitivity to cyanide inhibition of the oxidized enzyme [10]. In the crystal structures of CAT-1 and CAT-3 no amino acid modifications were apparent, besides the Cys-Tyr bond in CAT-1 (see below) [11,41]. However, partial modifications of Trp, His, or Met residues cannot be excluded, nor a modification at the mobile N-terminal loop that is not visible in the crystal structure of CAT-1 and CAT-3.

### Modification of the Tyr That Ligated the Fe of the Heme at the Proximal Side

The Tyr residue that ligates the heme Fe^III^ at the proximal side of the active site forms a covalent bond with a vicinal residue, a His in HPII [126] and a Cys in CAT-1 [41]. Catalase activity is required for this modification [126]. There is no reason to suppose that this modification is related to the heme *b* oxidation into heme *d* and, in fact, reactions are independent [59]. We have proposed a mechanism that explains how this covalent bond is generated [41]: the proximal Y379 of CAT-1 can give an electron to Cpd I to form a Cpd I* and a stabilized proximal tyrosine radical. Upon reduction by H_2_O_2_ of Cpd I*, O2^•−^ is liberated and the Fe^III^ porphyrin is restored. The tyrosine radical induces deprotonation of the sulfhydryl of the vicinal C356, and the Y379 is reduced by the thiolate forming a thiyl radical. Repeating the sequence of events, a covalent bond between the C-β atom of Y379 and the sulfur of the C356 is formed, and a second molecule of O_2_^•−^ is released. A covalent bond with the vicinal residue would make the Tyr less prone to donate an electron to Cpd I, turning these catalases resistant to H_2_O_2_ inhibition, in contrast to SSCs. Almost all L1–LSCs catalases have a Cys as the vicinal residue to the proximal Tyr, and these enzymes will probably form a covalent bond.

Instead of the Cys of the L1–LSCs (CAT-1), the PVC, CAT-3, CATPO, and almost all L2–LSCs have a Gln as the vicinal residue to the proximal Tyr. In CAT-3 (Q366) and PVC (Q393) this residue is oriented away from the proximal Tyr [11]. Thus, no covalent bond between the proximal Tyr and a vicinal amino acid residue is possible in L2–LSCs. However, the PVC also does not form Cpd I* [63]. Almost all L2–LSCs have the His required for the electron relay proposed in the HEC enzyme to fine-tune electron density at the active site [47]. Although the three amino acid residues required for the electron relay (Arg385, His249, and Asp379 in CAT-3) are conserved in most catalases, the L1–LSCs have an Asn instead of His. It is conceivable, but requires confirmation, that the proposed electron relay could avoid the formation of a radical at the proximal tyrosine.

## 8. What Is the Origin and Function of the TD in LSCs?

The presence of an additional TD in LSCs has been a mystery for many years. LSC was considered the archetypal monofunctional catalase from which SSC originated from gene duplication and loss of the TD [17]. However, it is unclear why LSCs were preserved in bacteria and fungi since the TD is not required for catalase activity. Moreover, in bacteria, a LSC has an important role in the stationary growth phase and spore germination; in filamentous fungi, LSCs are the main catalase activities, and there is an L2-type enzyme for growth and an L1-type for stationary growth and asexual spore formation. The TD of LSCs has a conserved flavodoxin-like topology related to the type 1 glutamine amidotransferase family [127] and the DJ-1/PfpI superfamily [128]. While the catalase domain has a well-preserved amino acid sequence, the TD presents considerable variation [19]. It has been shown that the TD confers LSCs a great stability [114,129], and loss of the TD labilizes the enzyme [129,130,131].

The amino acid composition of the catalase domain and the TD is different [132]: the catalase domain has decreased hydrophobic, an altered ratio of hydrophilic (D/E, N/Q, T/S, R/K) and increased heterocyclic/aromatic amino acid residues (F,W,P); the TD has increased amounts of hydrophobic residues and lower numbers of hydrophilic and heterocyclic/aromatic amino acids. The catalytic domain, which is conserved at both primary sequence and structural levels, has an amino acid composition that is optimized to select H_2_O_2_ over water and, at the same time, preserve its stability by incorporating “order promoting” amino acid residues; the TD, which is only structurally conserved, has an amino acid composition similar to very stable proteins and confers high stability to LSCs [132].

**Figure 10:**
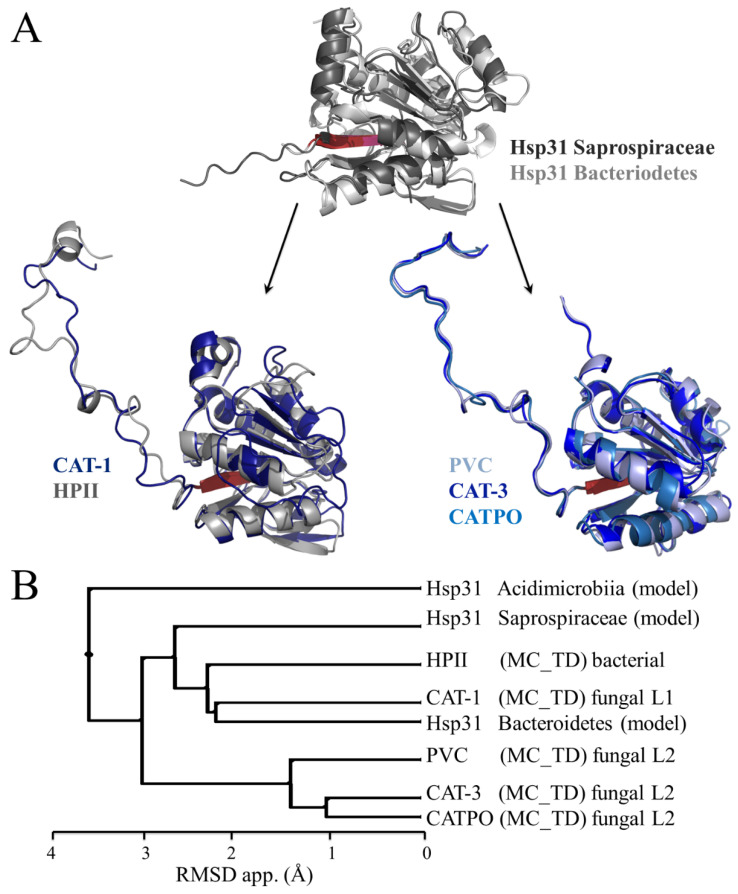
Structural alignment of bacterial Hsp31 proteins with the mobile coil–TD (MC_TD) region of LSCs. (**A**) Shown is the structural alignment of two bacterial Hsp31 (HchA) model-structures and the structural alignment of the MC_TD region of LSCs structures, CAT-1 and HPII (right), and CAT-3, PVC and CATPO (left). Bacterial structures are depicted in grey tones and fungal structures in blue tones. The first beta strand is shown in red. (**B**) Apparent RMSD of the structural alignment of three bacterial Hsp31 model-structures together with the MC_TD region of five LSCs. Bacterial Hsp31 model-structures downloaded from the AlphaFold Protein Structure Database: AF-A0A7Y1VWI9-F1-model_v3 DJ-1_PfpI family protein Saprospiraceae bact.pdb, AF-A0A7Y2ITD0-F1-model_v3 Protein deglycase HchA Acidimicrobiia bact.pdb, and AF-A0A7Y8NWU6-F1-model_v3 DJ-1_PfpI family protein Bacteroidetes bact.pdb.

Primary sequence and structural analysis with members of the DJ-1/PfpI superfamily indicates that the probable origin of the TD is a bacterial Hsp31: bacterial Hsp31 has an N-terminal loop that is not present in the Hsp31 proteins of other organisms, and the mobile coil that joints the catalase domain with the TD in LSCs has sequence similarity with the N-terminal loop of bacterial Hsp31 [132]. The structural analysis indicates that Hsp31 and DJ-1 proteins are more similar to the bacterial and fungal TDs than the other proteins of the DJ-1/PfpI superfamily. A phylogenetic analysis indicates that the bacterial Hsp31 sequences that consistently associate with the bacterial and fungal sequences of the mobile coil and the TD are from bacteria that live in marine extreme habitats and belong to different phyla. Three of these bacterial Hsp31 (HchA) (also labeled as DJ-1/PfpI family protein), downloaded from the AlphaFold Protein Structure Database, were structurally aligned with HPII, CAT-1, and three L2-type LSCs to determine their structural similarity (Figure 10). Remarkably, the Bacteroidetes Hsp31 has a very similar structure to the TD of CAT-1 (RMSD 2.2 Å), a fungal L1-type enzyme. The TD of CAT-1 is also very similar to the one of HPII.

The interpretation of these results is that LSC derived from a fusion between a bacterial Hsp31 gene and an SSC gene, which probably occurred very early in the bacterial phylogeny before the diversification of extant phyla [132]. This is consistent with the fact that LSCs are present in all phyla and that clade-1 SSCs appeared before LSCs [20].

Hsp31 is a well-known molecular chaperone present in most cells. The TD of LSCs, besides conferring stability to the catalase domain, has a chaperone activity that prevents the denaturation of other proteins, by heat, urea, or H_2_O_2_ [133]. The CAT-3 TD (TDC3) also protects SSCs from heat denaturation. SSCs and LSCs without the TD lack molecular chaperone activity. CAT-3 or TDC3 increases the survival of *E. coli* under heat or oxidative stress conditions, while the CAT-3 without the TD does not [133]. Localization of the chaperone activity is under way by performing deletions and amino acid substitutions in a hydrophobic and charged region of the TD.

The appearance of LSC conferred two great advantages for bacterial survival: a great stability to the catalase domain, which became resistant to different stress conditions, and a molecular chaperone activity that is particularly needed during stress to preserve the native active conformation of proteins including SSCs.

## 9. Concluding Remarks

Despite the existence of extensive literature on monofunctional catalases, the information about these enzymes is still fragmentary, and there are many issues that deserve examination and comprehension. More structural information on enzyme variants, MD studies, and density functional theory analysis are desirable. Control experiments for the new functions ascribed to catalase are needed. From the list of unanswered questions stated 20 years ago [44,134], there have been advances in the understanding of the mechanisms of the catalase reaction, the reactivity of Cpd I and the role of bound NADPH, how H_2_O_2_ reaches the active site, and on the origin and function of the TD of LSCs. However, in the last years the interest in elucidating the remaining questions related to catalases has been less intense. After a peak in 2008 in the number of papers published, articles on catalase investigations have diminished greatly. I hope that this review can contribute to renewing the interest in the research of these fascinating enzymes.

## Figures and Tables

**Table 1:** Catalase structures and their characteristics.

Name	Organism	Type	Clade	Heme Type	Heme Orient.	NADPH Binding				PDB
						217		203	305	
katA *	*Corynebacterium glutamicum*	SSC	3	*b*	III	S	+	R	Q	4B7G
MLC	*Micrococcus lysodeikticus* (*luteus*)	SSC	3	*b*	III	S	+	R	Q	1HBZ, 1GWF
KLC	*Kluyveromyces lactis*	SSC	3	*b*	III	G	+	R	H	6RJR
SCC-A	*Saccharomyces cerevisiae*	SSC	3	*b*	III	G	+	R	Q	1A4E
KPC	*Komagataella* (*Pichia*) *pastoris*	SSC	3	*b*	III	G	+	R	H	6RJN
PAC	*Hansenula polymorpha* (*Pichia angusta*)	SSC	3	*b*	III	G	+	R	H	2XQ1
HEC	*Homo sapiens* (erythrocyte)	SSC	3	*b*	III	S	+	R	H	1F4J
BLC	*Bos taurus* (liver)	SSC	3	*b*	III	S	+	R	H	3RE8
EFC	*Enterococcus faecalis*	SSC	3	*b*	III	S	+	R	Q	1SI8
HPC	*Helicobacter pylori*	SSC	3	*b*	III	S	-	R	L	1QWL, 2A9E
VSC	*Vibrio salmonicida*	SSC	3	*b*	III	S	+	R	H	2ISA
KatA	*Pseudomonas aeruginosa*	SSC	3	*b*	III	S	+	R	H	4E37
PMC	*Proteus mirabilis*	SSC	3	*b*	III	S	+	R	H	1M85, 1MQF, 1H7K
Cat-F	*Pseudomonas syringae*	SSC	1	*b*	IV	V	-	E	--	1M7S
DR1998	*Deinococcus radiodurans*	SSC	1	*b*	IV	V	-	W	R	4CAB
BPC	*Bacillus pumilus*	SSC	1	*b*	IV	V	-	E	E	4QOQ
EKTA	*Exiguobacterium oxidotolerans*	SSC	1	*b*	IV	V	-	E	E	2J2M
HPII	*Escherichia coli*	LSC	2	*d*	IV	I	-	R	E	1IPH, 1GGF, 1YE9
CAT-1	*Neurospora crassa*	L1-LSC	2	*d*	IV	V	-	R	E	3EJ6
CAT-3	*Neurospora crassa*	L2-LSC	2	*b*	IV	I	-	N	E	1SY7, 4AJ9
PVC	*Penicillium janthinellum* (*vitale*)	L2-LSC	2	*d*	IV	V	-	H	E	4CAT, 2IUF, 2XF2
CATPO	*Mycothermus thermophilus* (*Scytalidium thermophilum)*	L2-LSC	2	*d*	IV	V	-	H	E	5ZZ1

* gene name.

## Data Availability

Data is contained within the article.
